# Supplementary material for: Ionic imbalance induced self-propulsion of liquid metals
Source: Nat Commun. 2016 Aug 4;7:12402. doi: 10.1038/ncomms12402 (PMC4976217; doi:10.1038/ncomms12402)
Supplement: Supplementary Information — Supplementary Figures 1-9, Supplementary Tables 1-2, Supplementary Notes 1-2 and Supplementary References [file ncomms12402-s1.pdf]

Supplementary Figures

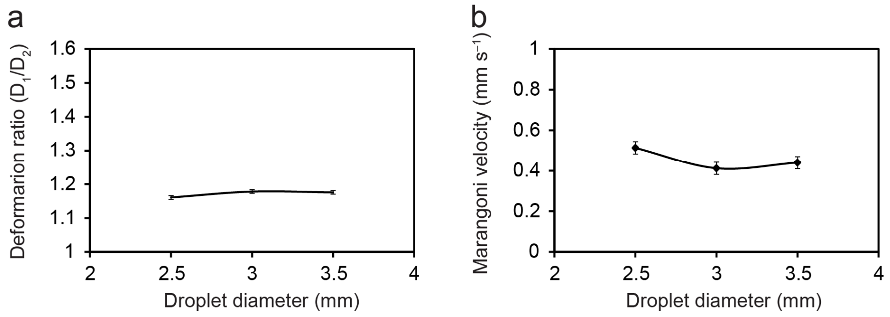

Supplementary Figure 1: Effect of the droplet size on droplet dynamics. (**a** and **b**) In three sets of experiments with varying droplet diameter, dynamics of the droplet in deformation dominant regions did not change significantly. Molarities are constant at 2.4 mol L^{-1} acid and 0.3 mol L^{-1} of base. Error bars indicated s.e.m. (n=6).

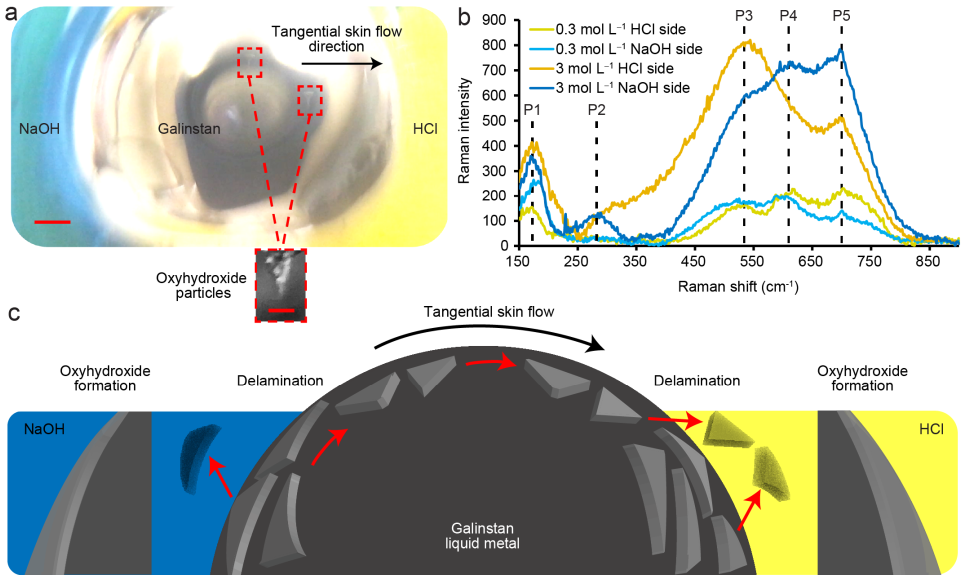

Supplementary Figure 2: Galinstan surface oxidization in high molarities of acid and base. (**a**) At high HCl and NaOH concentrations, relatively large and thick oxides flakes are formed on the surface of droplets and delaminated. These flakes move along the surface of Galinstan droplet toward the acidic side. The oxide flakes can be mechanically exfoliated simply by tweezers (inset). Scale bars are 0.5 and 0.1 mm in **a** and inset respectively. (**b**) To understand the composition and nature of the oxide flakes, the surface of the two sides of the droplet were analyzed using Raman microspectroscopy. Results indicate five distinct peaks (P1-5) matching Raman spectra of α-gallium oxyhydroxide[1]. The peaks' intensities increased significantly when droplet was exposed to higher imbalanced concentrations, which indicates the formation of much thicker α-gallium oxyhydroxide flakes (at least 4-5 times thicker comparing 0.3 and 3 mol L^{-1} concentrations if the relation is considered linear). Peak intensities are different for skins in NaOH and HCl areas due to the difference of hydration and surface charges. Due to the damping effect of liquid metal under the surface of flakes, peaks located in wavenumbers lower than 500 cm^{-1} are weakened. (**c**) In higher molarities of electrolytes, thicker layers of α-gallium oxyhydroxide flakes form on the NaOH side of the droplet. These thick layers delaminate from the surface, with the help of bubbles that are produced by the chemical reactions on the surface of droplet and move toward the HCl side under the effect of surface tension difference. This causes a tangential skin flow. These flakes are eventually released into the aqueous solution as the effect of the flow in the channels.

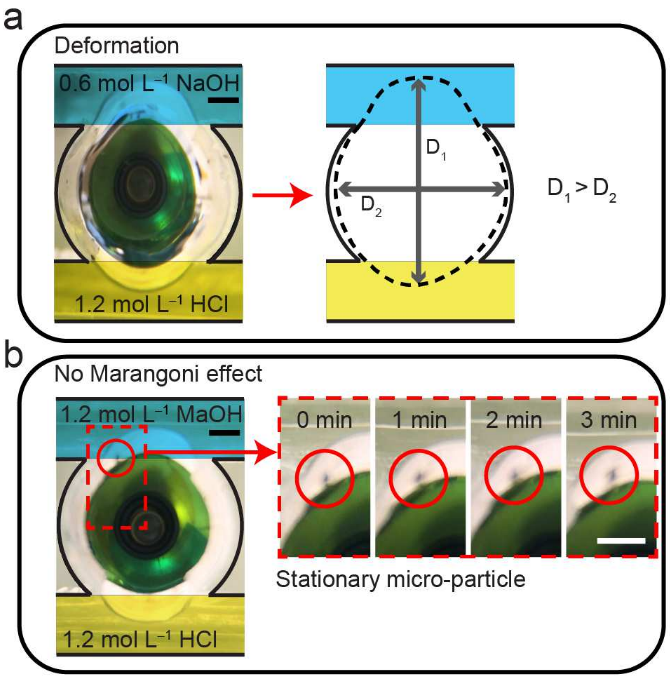

Supplementary Figure 3: Mercury droplet dynamics under ionic imbalance. (**a**) At low concentrations of electrolyte, droplet deformation is observed, similar to that of Galinstan. (**b**) At high concentrations of electrolytes, the mercury droplet does not exhibit any tangential skin flow due to Marangoni effect, as opposed to the Galinstan liquid metal. This is evidenced as the micro particles deposited over the surface of mercury droplet remain stationary. Scale bars are 0.5 mm.

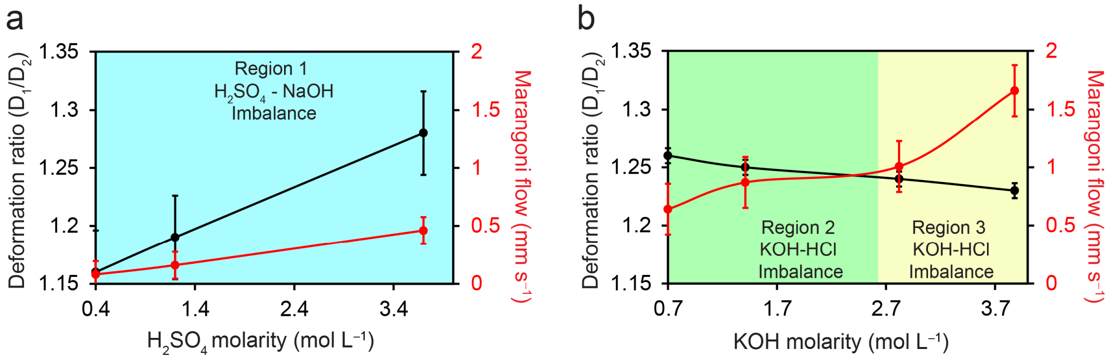

Supplementary Figure 4: Effect of pH imbalance of different acid and base solutions on droplet dynamics. (**a**) Varying concentration of H_2SO_4 is used with constant 0.3 mol L^{-1} of NaOH. As expected, droplet deformation was more dominant. Background is colored in blue to indicate the droplet is operating in region 1 of the reference diagram of Fig. 2D. (**b**) Region 2 and 3 are tested with varying concentration of KOH and constant concentration of 3.4 mol L^{-1} HCl. The Marangoni dominant region is shifted to higher concentrations of KOH solution than that of NaOH.

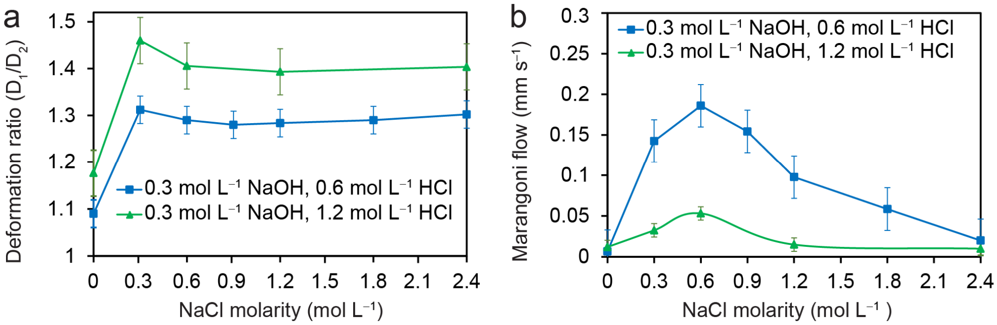

Supplementary Figure 5: Droplet dynamics under imbalanced mix ionic solution of low NaOH molarity. NaCl mixed solutions are prepared in low molarities of 0.3 mol L^{-1} NaOH. HCl is kept constant at 0.6 mol L^{-1}. Error bars indicated s.e.m. (n=6). (**a** and **b**) Graphs present deformation ratio and Marangoni flow in varying concentrations of NaCl while concentrations of NaOH and HCl are kept constant. NaCl is mixed with NaOH in all experiments. In **a** Maximum deformation is seen in 0.3 mol L^{-1} of NaCl while Marangoni flow maximum in **b** is located at 0.6 mol L^{-1} of NaCl.

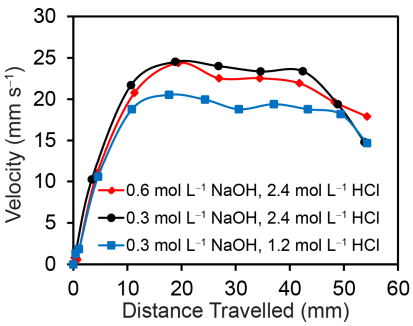

Supplementary Figure 6: Self-propelling droplet with instantaneous velocity. Graph of three experimental velocity profiles with droplet velocities exceeding 20 mm s^{-1}.

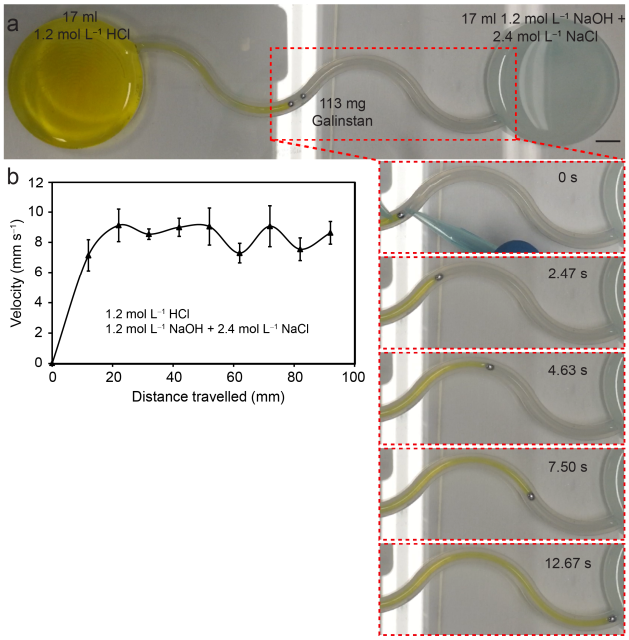

Supplementary Figure 7: Channel Configuration to obtain high velocity profile of the self-propelling droplet. (**a**) Self-propelling droplet was tested in a longer serpentine channel. Configuration has total channel length of 18.3 cm and reservoir sizes of 5.5 cm. (Supplementary Movie 5). Experiment is conducted as explained in experiment 3 methods. (**b**) Configuration is selected from region 2 of Fig. 2c to demonstrate the velocity profile in a region with measurable Marangoni flow and less deformation ratio. The droplet velocity profile shows a cyclic behavior and descends regularly due to the mixing effect caused by the Marangoni flow.

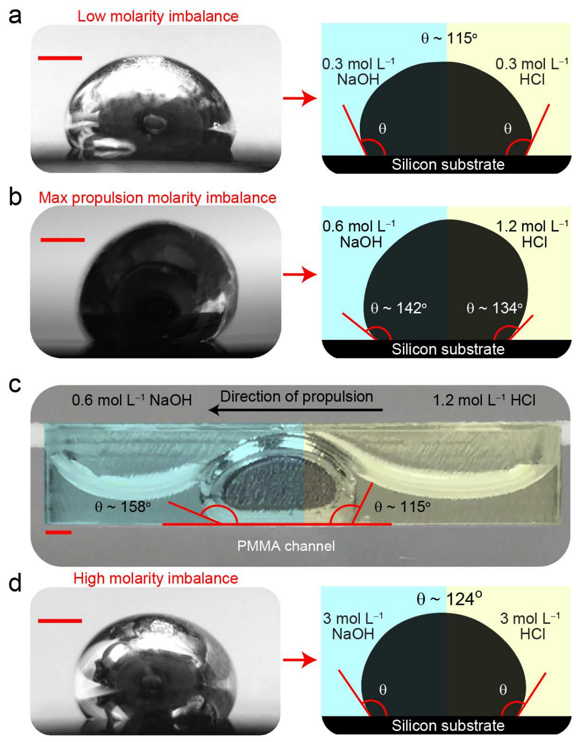

Supplementary Figure 8: Droplet contact angle. (**a**) Droplet contact angle on a silicon substrate after the exposure to relatively low molarity imbalance, which results in soft and deformable skin. (**b**) Droplet contact angle after the exposure to molarities, which produce the maximum propulsion. (**c**) Droplet showing asymmetric contact angles when placed in ionic imbalance condition in **b**. (**d**) Droplet contact angle after the exposure to relatively high molarity imbalance which results in contact angles for producing less wettability and deformability than that of **a**. **d** represents an example of the Marangoni region.

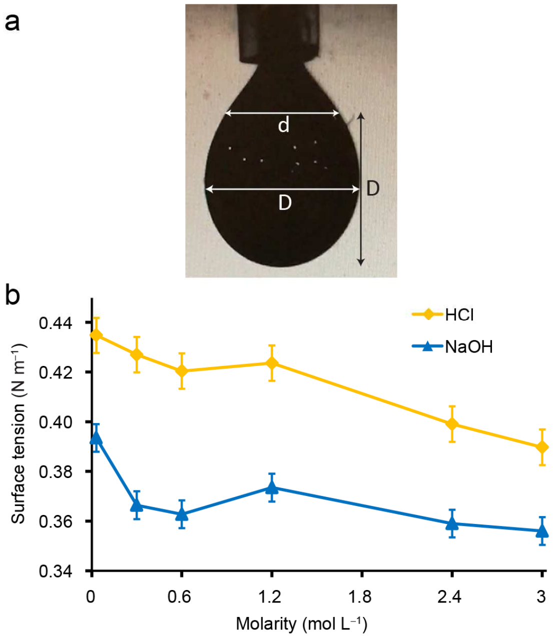

Supplementary Figure 9: Surface tension measurement between Galinstan and aqueous ionic electrolytes. (**a**) Surface tension was measured using pendant drop method[2]. (**b**) Surface tension of Galinstan in contact with aqueous solutions of different HCl and NaOH concentrations. Error bars are s.e.m. (N=4).

Supplementary Tables

Supplementary Table 1: Ionic coefficients

	1.2 mol L^{-1} HCl	0.6 mol L^{-1} HCl	0.3 mol L^{-1} HCl + 0.3 mol L^{-1} NaCl	0.3 mol L^{-1} NaOH + 0.3 mol L^{-1} NaCl	0.6 mol L^{-1} NaOH
α (mm/v s^{-1})	1.8218 (R^2 =0.93)	6.3857 (R^2 = 0.89)	3.9984 (R^2 =0.98)	0.3048 (R^2 = 0.89)	0.2621 (R^2 = 0.94)
φ_{Th} (v)	1	0.8	1	1.6	1.7
φ_0 (v)	1.4	1.5	1.7	1.9	2.1

List of experimental measurements of the Marangoni flow coefficient α, threshold potential φ_{Th} and potential of zero charge φ_0. R^2 is the coefficient of determination of the linear regression trend line gradient of the Marangoni flow velocity against potential.

Supplementary Table 2: Values of A and B_i for pendant drop shape analysis

Range of S	A	B_4	B_3	B_2	B_1	B_0
0.401-0.46	2.56651	0.32720	0	0.97553	0.84059	0.18069
0.46-0.59	2.59725	0.31968	0	0.46898	0.50059	0.13261
0.59-0.68	2.62435	0.31522	0	0.11714	0.15756	0.05285
0.68-0.90	2.64267	0.31345	0	0.09155	0.14701	0.05877
0.90-1.00	2.84636	0.30715	−0.69116	−1.08315	−0.18341	0.20970

Supplementary Notes

Supplementary Note 1: Energy calculations of the surface Marangoni flow

Induced Marangoni flow of the liquid metal was measured on the surface of the droplet at different applied potentials and concentrations of ionic solution. In order to calculate the surface tension gradient, which causes the Marangoni flow, the kinetic energy of the flow is calculated first. We assume negligible viscous losses at the air/droplet interface. In Marangoni dominant regions, the droplet is approximately spherical $\frac{D_1}{D_2} \cong \frac{R}{R} = 1$ (in which R is droplet radius) then:

$$E_{Unit\ Area} = \frac{\partial E_{Kinetics}}{\partial A_s} = \frac{1}{2}\left(\frac{\partial m_s u_s^2}{\partial A_s}\right) = \frac{1}{2}\left(\frac{\partial A_s \delta \rho_s u_s^2}{\partial A_s}\right) = \frac{1}{2}\delta \rho_s u_s^2 \qquad (1)$$

where $\partial E_{Unit\ Area}$ denotes the kinetic energy of the Marangoni flow per area on the surface of the droplet, m_s is the displaced mass (this mass is made of surface liquid of the Galinstan, liquid in its vicinity and delaminated skin oxide layer in Fig. 2b and Supplementary Information Fig. 2c), U_s is the skin tangential flow velocity generated by the Marangoni effect, A_s is the surface area of the moving layer, ρ_s is the density of the displaced mass, δ is the effective thickness of the displaced layer (Fig. 2b) at the interface of liquid metal (which also includes the surface oxide skin)

From the experimental results and measurements, Marangoni flow is constant over the surface and proportional to the potential difference as:

$$u \propto (\varphi - \varphi_{Th}) \ \rightarrow \ u = \alpha(\varphi - \varphi_{Th}) \qquad (2)$$

in which φ is the applied potential, φ_{Th} is the threshold potential for each ionic liquid, at which Marangoni flow commences, and α is a constant number that is defined as the ionic liquid gradient of Marangoni flow graph against potential listed in Table S1.

The droplet resides in a circular recess. Therefore, the liquid metal at the interface with the recess has a zero velocity and surface Marangoni flow occurs predominantly on the surface of the top hemisphere of the droplet. Combining (1) and (2) result in:

$$E_{Unit\ Area} = \frac{1}{2}\delta\rho_s\alpha^2(\varphi - \varphi_{Th})^2 \qquad (3)$$

which is only valid for $\varphi > \varphi_{Th}$. Consequently, the surface tension changes, accordingly the Marangoni Flow effect can be approximated as:

$$\Delta\gamma_{Marangoni\ Flow} \cong -\frac{1}{2}\delta\rho_s\alpha^2(\varphi - \varphi_{Th})^2 \qquad (4)$$

Supplementary Note 2: Surface tension measurement between Galinstan and aqueous ionic electrolytes

Pendant drop shape analysis method is used for measuring interfacial tension between aqueous solutions and liquid metal droplet. The shape of the droplet is governed by the balance between gravitational and surface tension forces, from which the interfacial tension of droplet-liquid is obtained as:

$$\gamma = (\triangle\, \rho\, g\, D^2)/H \tag{5}$$

where $\triangle\, \rho$ is the difference in fluid densities, g is the gravitational acceleration, D is the equatorial diameter, and H is a shape dependent parameter, which is obtained using equation (2):[2]

$$\frac{1}{H} = \frac{B_4}{S^A} + B_3S^3 - B_2S^2 + B_1S - B_0 \tag{6}$$

in which, "$S=d/D$" is the shape factor with d defined as the diameter of droplet at the distance D from the bottom of the droplet. The values of A and B_i (i=0, 1, 2, 3, 4) are empirical constants, as given in Supplementary Table 2[2].

Supplementary References

1 Zhao, Y., Yang, J. & Frost, R. L. Raman spectroscopy of the transition of α-gallium oxyhydroxide to β-gallium oxide nanorods. *J. Raman Spectrosc.* **39**, 1327-1331 (2008).

2 J. Drelich, C. F., and C. L. White. *Measurement of interfacial tension in fluid/fluid systems*. Ch. Measurement of Interfacial Tension in Fluid-Fluid Systems, 3152-3166 (Marcel Dekker Inc, New York, U.S.A., 2002).
